# Case Report of Rare Type Submucosal Polyp–Angiolipofibroma of Sigmoid Colon

**DOI:** 10.1155/2019/1896275

**Published:** 2019-03-19

**Authors:** Khin San Aye, Aung Khine Zan, Myo Thet Tin, Than Than Aye

**Affiliations:** ^1^Department of Gastroenterology, University of Medicine 2, Yangon, Myanmar; ^2^Yangon General Hospital, Myanmar; ^3^Nay Pyi Taw General Hospital (1000-bedded), Myanmar

## Abstract

Most of the colonic submucosal mesenchymal polyps are benign tumors. They are formed by more than one type of mesenchymal tissue. The diagnosis of angiolipofibroma depends on the histological findings with the proliferation of vascular, fatty, and fibrous connective tissues. Majority of angiolipofibromas are arising in the kidney and have rare presentation as extra renal region. Here we report a case of 58-year-old female patient with angiolipofibroma of sigmoid colon (8.5 cm x 4 cm size) which was treated successfully with surgical resection. The clinical presentation, operative procedure, pathological features, and medical literature review are presented.

## 1. Introduction

Most of the colonic polyps are originated in the mucosa and a small subset of them originate from the submucosal mesenchymal. The most common types of submucosal polyp are composed of single type tissue include lipoma, leiomyoma, neurofibroma or mucosal Schwann cell hamartomas, vascular lesion, and gastrointestinal stromal tumor (GISTs) [1]. The mixed mesenchymal lesions are extremely rare. Angiolipofibromas are a form of mesenchymal hamartoma, composed of more than single type tissue including blood vessels, smooth muscle cells, and mature fat cells. Majority of angiolipofibromas arise in the kidney and extrarenal angiolipofibromas have rare presentations [2]. Again, colonic angiolipofibroma is rare benign tumors of the extra renal type with particular histopathologic characteristics. 

The aim of this report is to present an unusual case of colonic polyp originating from submucosal with mixed mesenchymal composed of vascular, mature fatty, and fibrous connective tissues.

## 2. Case Presentation

A 58-year-old female patient presented with one episode of rounded mass protruded from anus during defecation and reduced manually by herself. She was previously healthy person and had normal bowel motion. She had no history of abdominal pain or mass, bleeding per rectum, and weight loss. There was no family history of colonic polyps or colon cancer. On physical examination, there was no significant anemia, no palpable abdominal mass nor tenderness. Stool examination was positive for occult blood. Her hemoglobin was mildly reduced 11.6 gm/dl with hypochromic microcystic type of anemia and a serum carcinoembryonic antigen level (0.32 ng/ml) was normal. Other laboratory tests like blood sugar, renal profiles, and liver profiles were within normal ranges.

**Figure 1:**
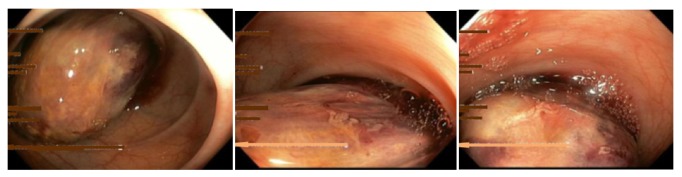
Endoscopic finding.

**Figure 2:**
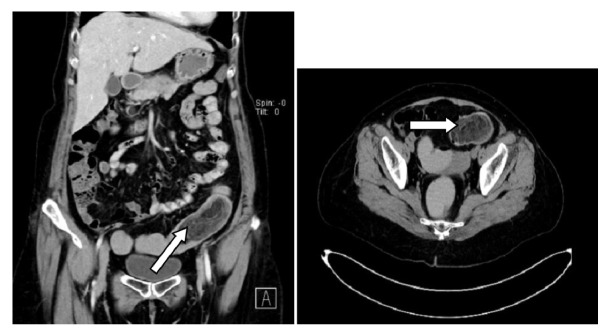
CT abdomen.

A full colonoscopy was performed. A large polyp about 8 cm with rather smooth surface but highly vascular was found at the sigmoid colon, 14 cm from anal verge. The mass was occupied two third circumference of whole colonic lumen (Figure 1). The origin of the polyp or stalk was difficult to identify. CT abdomen was done before sent for surgery. The finding was a circumferential well defined arterial contrast enhancing bowel mass measurement of 8.1 cm length arising from the distal part of the descending colon. Entrapped fat attenuations were seen inside the colonic lumen. No mesenteric adenopathy was seen. CT diagnosis was fat containing mesenchymal tumor (GISTS) of descending colon (Figure 2). Patient was planned for surgical resection without biopsy because of risk of hemorrhage.

**Figure 3:**
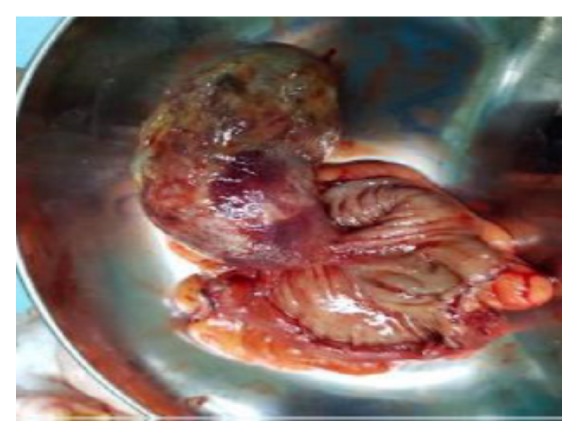
Gross surgical section of polyp.

In laparotomy, a large polypoid lesion about 8 x 3 cm with short stalk was found in sigmoid colon. The serosal surface appeared normal and no adjacent intra-abdominal lymph nodes were enlarged. Affected portion of sigmoid colon was resected and sent for histology examination (Figure 3). No renal mass was seen grossly during operation.

## 3. Pathologic Report

**Figure 4:**
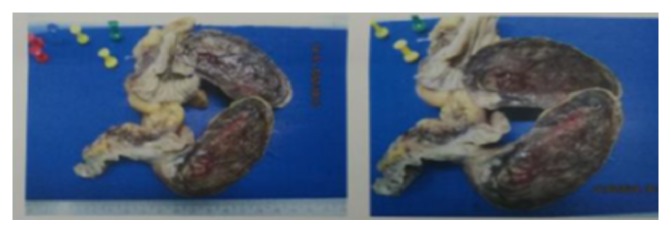
Gross pathological section of polyp.

Macroscopic examination of the excised specimen showed a polypoid growth measured 14 x 9 x 5 cm, of soft consistency, covered by grayish white fleshy mucosa. The luminal surface of polypoidal growth was measured 8.5 x 4 cm. The mucosa surface appeared normal. No hemorrhagic or necrosis was seen (Figure 4).

**Figure 5:**
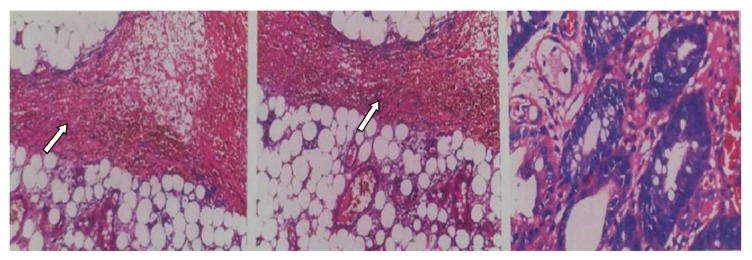
Histological finding of polyp.

Histological finding was large pedunculated polyp arising from the wall and composed of mature adipocytes and proliferating large and small vessels of varying nature (capillaries, small veins, venules, arterioles, and small arteries) lined by a thin wall endothelial lining. No vasculitis or thrombosis was noted. There also proliferated smooth muscle spindle cells with eosinophilic cytoplasm embedded in loose myxoid stroma. Histological diagnosis was benign angiolipofibroma polyp, sigmoid colon (Figure 5).

## 4. Discussion

Colonoscopy is the gold standard procedure for colonic screening of colorectal cancer or polyps [3]. In this case, we found submucosal polyp showing a characteristic histological triad of adipose tissue, vessels, and smooth muscle of mixed mesenchymal structures normally present in the submucosa supporting the diagnosis of an angiolipofibroma hamartoma rather than a true neoplasm.

The majority of angiolipofibromas are benign tumors derived from mesenchymal tissue and majorities are arising in the kidney. Extra renal presentations are very rare and reported to be found in the liver, nasal cavity, vagina, spermatic cord, skin, mediastinum, and GI tract. Among GI tract involvement, colonic involvement is rather rare condition [2].

Angiolipofibroma may be a challenging in diagnosis when it arises outside its typical renal location because of its rarity and the histological variation. Typically, angiolipofibroma shows a mixture of adipose tissue, fat, and abnormal vessels, but it may show a wide range of histological appearances. These include tumors where the muscle component is dominant, including epithelioid variants band showing considerable nuclear atypia. Preoperative diagnosis of angiolipofibroma is difficult [4].

Demir et al. described a similar lesion originating in the mesocolon of the sigmoid, composed of vascular proliferation in a fibrofatty tissue background; it was published in the name of “angio lipomatous mesenchymal hamartoma” [5]. Groisman et al. described a cecal submucosal polyp formed by adipose tissue, fibrous tissue, and vascular proliferation and lymph vessels in variable sizes under the name “angiolipofibroma of the cecum” [6]. Another common differential diagnosis is lipomatous neoplasms formed by adipose tissue and commonly found in the right colon [7]. Vascular lesions seen in the gastrointestinal tract formed solely by vascular proliferation include lymphangiomas, hemangiomas, hemangiolymphangiomas, and congenital vascular malformations [8].

Only few cases of colonic angiolipofibroma have been reported in the literature [9–11]. Most of them occurred in middle aged men. The natures of the polyps were polypoid or pedunculated nature and located in the colon mostly on the left sided. Most of the colonic angiolipofibromas are presented as small polyps and can successfully be removed by endoscopically and rarely need surgery, but this case presented as a large lesion and needed surgery.

As the recurrence of colonic polyps, it is known that adenoma recurrence rates are estimated to be around 30-40%, 3 to 4 years after the initial colonoscopy [12, 13]. The risk of recurrent adenomas on the surveillance colonoscopy depends on the findings at the initial diagnosis. The risk is higher in advanced and/or multiple adenomas [14, 15]. Some study mentions the recurrence rate of polyp on left colon and rectum is of higher rate than that on right colon. But there remains controversial whether the location may affect the recurrence of polyps [16]. Although there was no data about recurrent rate of colonic angiolipofibroma after resection, we do follow up this patient as usual postpolypectomy followup. Patient is planning to do recheck colonoscopy and CT abdomen after one year and ultrasound abdomen every six months. Our patient is well and uneventful during followup of 6 months and no renal mass and other pathologies are seen in ultrasound examination.

## 5. Conclusion

This submucosal mesenchymal, angiolipofibroma polyp case was reported on left side of the colon as the common location but differed from others, which was larger polyp (8.5 x 4 cm) with short or no stalk not favored to be removed endoscopically. Surgical excision is the treatment of choice for large lesion.
